# Unraveling the celiac disease-related immunogenic complexes in a set of wheat and tritordeum genotypes: implications for low-gluten precision breeding in cereal crops

**DOI:** 10.3389/fpls.2023.1171882

**Published:** 2023-05-11

**Authors:** Miriam Marín-Sanz, Francisco Barro, Susana Sánchez-León

**Affiliations:** Department of Plant Breeding, Institute of Sustainable Agriculture (IAS), Spanish National Research Council (CSIC), Córdoba, Spain

**Keywords:** amplicon, alpha-gliadins, gamma-gliadins, precision breeding, wheat, CRiSPR/Cas

## Abstract

The development of low-gluten immunogenic cereal varieties is a suitable way to fight the increment of pathologies associated with the consumption of cereals. Although RNAi and CRISPR/Cas technologies were effective in providing low-gluten wheat, the regulatory framework, particularly in the European Union, is an obstacle to the short- or medium-term implementation of such lines. In the present work, we carried out a high throughput amplicon sequencing of two highly immunogenic complexes of wheat gliadins in a set of bread and durum wheat, and tritordeum genotypes. Bread wheat genotypes harboring the 1BL/1RS translocation were included in the analysis and their amplicons successfully identified. The number of CD epitopes and their abundances were determined in the alpha- and gamma-gliadin amplicons, including 40k-γ-secalin ones. Bread wheat genotypes not containing the 1BL/1RS translocation showed a higher average number of both alpha- and gamma-gliadin epitopes than those containing such translocation. Interestingly, alpha-gliadin amplicons not containing CD epitopes accounted for the highest abundance (around 53%), and the alpha- and gamma-gliadin amplicons with the highest number of epitopes were present in the D-subgenome. The durum wheat and tritordeum genotypes showed the lowest number of alpha- and gamma-gliadin CD epitopes. Our results allow progress in unraveling the immunogenic complexes of alpha- and gamma-gliadins and can contribute to the development of low-immunogenic varieties within precision breeding programs, by crossing or by CRISPR/Cas gene editing.

## 1 Introduction

Bread wheat (*Triticum aestivum* L.) is the dominant crop in temperate countries widely used in the human diet and livestock feed. It is an essential source of nutrients and other beneficial compounds. World production of this crop reaches 760 million tons per year, representing 30% of all harvested cereals (FAO, 2022). Wheat grains contain around 9-15% of protein, and derived wheat flour is processed into a great variety of food products that humans consume daily, such as bread, cakes, noodles, biscuits, etc. Grain proteins can be classified according to their extractability in a series of solvents: albumins (soluble in water), globulins (soluble in salt), gliadins (soluble in alcohol), and glutelins (insoluble in other solvents and extracted in alkali) (Osborne, 1907). In wheat, glutelins are mainly composed of glutenins which are, in fact, prolamin subunits not extractable with alcohol/water solvents due their polymeric nature by inter-chain disulphide bonds (Shewry, 2019). Both groups, gliadins and glutenins, comprise gluten, and they are also divided into different fractions; the first one into three structural groups: omega-, alpha-, and gamma-gliadins; and the second one into two types of subunits: the High Molecular Weight (HMW), and the Low Molecular Weight (LMW) glutenin subunits (Shewry, 2009). Despite the ability to provide breadmaking properties, these proteins present epitopes that can trigger the immune response in patients suffering from celiac disease (CD), an autoimmune enteropathy provoked by the presence of certain gluten peptides which are deamidated by the tissue transglutaminase 2 (tTG2) in the human gut. These peptides are presented by the human leukocyte antigen (HLA)-DQ2 and DQ8 haplotypes to the CD4 T cells, triggering the immunogenic response in the small intestine and leading to a cascade of symptoms like intestine inflammation, the atrophy of intestinal villus, and malabsorption of nutrients, among others (Sollid, 2002; Ludvigsson et al., 2013). In a recent meta-analysis, the global prevalence of CD was 0.7% in biopsy-confirmed patients, reaching values of 2-3% in countries such as Finland and Sweden (Catassi et al., 2014; Singh et al., 2018).

However, not all the HLA-DQ molecules are linked to the same level of risk. The HLA-DQ2.5 molecule binds to more gluten peptides compared to HLA-DQ2.2 and forms stable complexes with the gluten T-cell epitopes (Vader et al., 2003; Fallang et al., 2009). Over the years, many HLA-DQ2.5 epitopes have been characterized in gluten proteins; showing that they are mainly present in the gliadin fraction of gluten (Sollid et al., 2012; Sollid et al., 2020). Particularly, the alpha-gliadins of wheat contain the 33-mer peptide, one of the most immunogenic peptides known so far. This peptide contains six overlapping copies of three epitopes and shows high stimulatory properties in relation to CD (Shan et al., 2002). In addition, the alpha-gliadins of wheat also present the p31-43 peptide, which is associated with the innate immune response in CD non-T cell-dependent (Maiuri et al., 2003). In the wheat gamma-gliadins, it was identified another highly immunogenic peptide denoted as 26-mer, which is also highly resistant to intestinal brush border membrane proteolysis and contains many overlapping epitopes (Shan et al., 2005).

In wheat, the alpha- and gamma-gliadin proteins are encoded by two multigene families in the *Gli-2* and *Gli-1* loci located, respectively, in the short arm of the homoeologous chromosome 6 (Huo et al., 2018b) and 1 (Anderson et al., 2012; Altenbach et al., 2020). Both loci contain a large number of duplicated genes arranged in tandem that, in addition to the wheat polyploid nature, the high content of repetitive DNA and the large size of the genome, make it difficult to obtain high-quality sequences for these gene families. In fact, for the alpha-gliadins between 25 to 150 copies in wheat cultivars and ancestral species were proposed (Harberd et al., 1985). Recently, a droplet digital PCR (ddPCR) assay revealed the copy number variation of different wheat genotypes, obtaining 70-76 copies of alpha-gliadins in tetraploid wheat (AABB), and 86-95 copies in synthetic hexaploid wheat genotypes (AABBDD) (Jouanin et al., 2020).

There is a high variation of the immunogenicity among wheat varieties (Spaenij–Dekking et al., 2005), which is tightly linked to the variation of CD-immunogenic epitopes present in the gluten proteins. The characterization of the epitope content and the identification of genotypes with low immunogenic potential is the starting point in precision breeding programs and would facilitate the development of non-genetically modified low-gluten wheat genotypes. In addition, these CD low-epitope genotypes could be the targets for genomic editing as their reduced structural complexity will facilitate the use of Cas enzymes to develop low-immunogenic genotypes.

In previous work, we analyzed di-, tetra-, and hexaploid wheat accessions by amplicon-based Next-Generation Sequencing (NGS) to study the origin and evolution of different types of alpha-gliadins (Ozuna et al., 2015). This amplicon expands the p31-43 and 33-mer, allowing the identification of both peptides along with the DQ2.5_glia_α3, which overlaps partially with the 33-mer peptide. This approach provided an excellent tool for addressing the structural complexity of the immunogenic alpha-gliadins, and for the selection of wheat varieties with very low CD immunogenic epitopes. In this work, we report the characterization of the gamma-gliadin immunogenic complex by NGS amplicon sequencing, and the utility of both alpha- and gamma-gliadins amplicons to study a wide range of cereal genotypes comprising bread wheat, durum wheat, and the amphiploid tritordeum (AABBH^ch^H^ch^), a fertile cross between the wild barley *Hordeum chilense* and durum wheat, which showed lower immunogenic properties than bread wheat (Haro et al., 2022). Based on their alpha- and gamma-gliadins epitopes abundance, we can anticipate their immunogenic potential concerning CD. More interestingly, we can identify wheat varieties containing rye translocations and their associated epitopes. Results reported in this work are important not only for selecting potential low-immunogenic cereals but also to select wheat varieties with low gliadin complexity, which can be used as targets for genome editing by CRISPR/Cas (Sánchez-León et al., 2018).

## 2 Materials and methods

### 2.1 Plant material

A total of 44 genotypes were analyzed comprising 37 bread wheat genotypes, two genotypesof durum wheat, and five genotypes of tritordeum (**Supplementary Table 1**). Among the bread wheat, 14 of them presented rye translocation (1BL/1RS translocation) as denoted by the presence of the allele *Gli-B1l* in the mature grains gliadin proteins profiles by A-PAGE (Metakovsky et al., 2018) (**Supplementary Figures 1, 2**). The protocol followed for A-PAGE gels of total gliadin proteins is described in Gil-Humanes et al. (2012). For the genotypes BIOINTA 2004 and INIA Condor, the allele A-PAGE protein profile was not as clear as the other bread wheat with rye translocation, but both presented amplicons annotated as 40k-γ-secalins from genes located in the 1RS chromosome of rye (Qi et al., 2013). Per duplicate, all the plants were grown in a greenhouse and the leaves were collected in the first stages of the plant and frozen at -80°C.

### 2.2 DNA extraction, PCR and NGS sequencing

**Figure 1:**
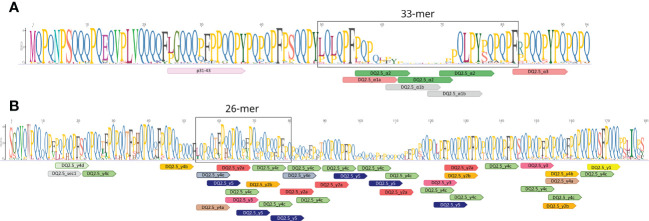
Schematic diagram of amplicon-encoded proteins of the **(A)** alpha and **(B)** gamma gliadins. The amplicons are located in the first repetitive domain of both gliadin proteins. Mapping of different DQ2.5 celiac disease (CD) epitopes were shown using the Geneious v2019.0.3 software (https://www.geneious.com). The consensus sequences of alpha- and gamma-gliadins from BW208 clones, the NCBI, and RefSeq v1.1 reference genome annotation of bread wheat were used for the representation.

The total DNA was extracted for each leaf sample following the CTAB protocol (Murray and Thompson, 1980). The DNA concentration was determined by NanoDrop ND-1000 (Thermo Fisher Scientific, Waltham, MA, United States). The primers aGli900F1 (5’-GTTAGAGTTCCAGTGCCACAA-3’) as forward and 33mer1R2-Ok (5’-GGTTGTTGTGGTTGCGRATA-3’) as reverse, and γgliF3 (5’-GCCAATATRCAGGTCGACCC-3’) as forward and γgliR3 (5’-GGGTTCAWCTGTTGTTGTAG-3’) as reverse were used to amplify the alpha and gamma-gliadin amplicons, respectively. The primers of the alpha-gliadin amplicon were previously designed by Ozuna et al. (2015) in a conserved region of the genes coding the first repetitive domain of the alpha-gliadin proteins which comprises the p31-43 region associated with the innate response in CD and the 33-mer peptide (**Figure 1A**). The primers for the gamma-gliadin amplicon were designed in the present work to amplify the gene sequence coding part of the N-terminal and the first repetitive domain of gamma-gliadin proteins comprising a high number of CD epitopes, including the 26-mer peptide (**Figure 1B**). These primers also amplified the 40k-γ-secalins (located in the short arm of chromosome 1R) which have high homology with gamma-gliadins of wheat (Qi et al., 2013). The amplicon sequencing was carried out by Fundación Parque Científico de Madrid (Cantoblanco, Madrid) by the MiSeq system (https://www.illumina.com) producing paired-end reads of 2x280 bp. The amplicon lengths were checked by the Agilent 2100 Bioanalyzer system (Agilent Technologies, Santa Clara, CA 95051).

### 2.3 Amplicon analysis

**Figure 2:**
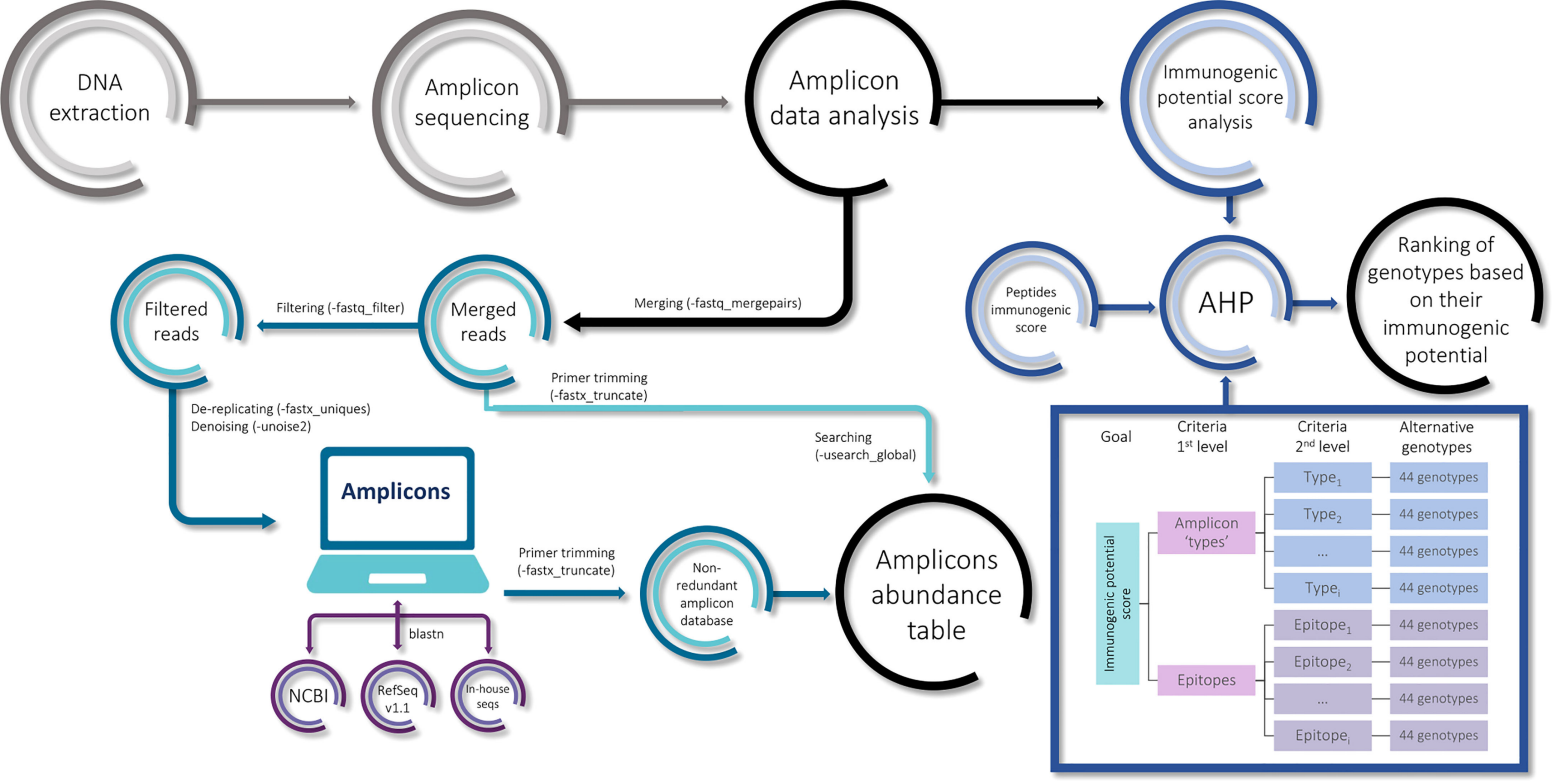
The pipeline of the amplicon analysis.

A GNU/Linux Ubuntu v 18.04 (https://ubuntu.com) server with 64 cores and 128 GB of RAM was used for sequences analysis. For the amplicon, the raw reads were analyzed by Usearch v9.2.64 (Edgar, 2010). Briefly, the raw paired-end reads were merged (-fastq_mergepairs), filtered (-fastq_filter), de-replicated (-fastx_uniques), and denoised (-unoise2) to obtain the unique denoised amplicons for all the genotypes (**Figure 2**). From now on, the unique denoised amplicons will be referred to as ‘amplicons’ for ease of reading. This protocol was run independently for alpha- and gamma-gliadins/secalins.

A non-redundant amplicon database was constructed for alpha- and gamma-gliadins separately. In addition, alpha- and gamma-gliadin gene sequences obtained from the National Center for Biotechnology Information (NCBI), the RefSeq v1.1 bread wheat reference genome annotation (Appels et al., 2018), and in-house sequences of the wheat variety BW208 clones by Sanger sequencing were included. The primers were aligned to these sequences to extract the corresponding amplicon sequence and, subsequently, those primers were removed from all the database entries by Usearch (-fastx_truncate) (**Figure 2**). For the gamma-gliadin amplicon analysis, seven 40k-γ-secalin sequences were included from the NCBI. The amplicons sequences obtained by Usearch were aligned in the NCBI database, the RefSeq v1.1, and the BW208 clones’ sequences by blastn (Johnson et al., 2008) to avoid redundancy. Unique sequences were kept for the alpha- and gamma-gliadin/secalin amplicon databases.

After primer trimming, the alpha- and gamma-gliadin/secalin reads for each genotype were searched in both databases respectively with an identity score of 0.99 (-usearch_global -id 0.99) to calculate the abundance of the amplicons. The amplicons with less than 0.25% of abundance in at least four samples were discarded for the analysis. The abundance of each amplicon was normalized by the total number of reads of the amplicons present in each genotype. The mean of the two biological replicates per genotype was calculated for further analyzes.

#### 2.3.1 Epitope search and amplicon types

For analyzing the potential immunogenicity of wheat and tritordeum genotypes based on the NGS data, the amplicons were translated to peptides for searching the non-deamidated CD epitopes in their sequence with Python custom scripts (https://github.com/MiriamMarinS/wheatAHP). As reported in Sollid et al. (2012), the DQ2.5_glia_ω1, DQ2.5_hor_1, and DQ2.5_sec1 share the same epitope sequence; like the DQ2.5_hor_2 and DQ2.5_sec2. Therefore, they were denoted as ‘sec1’ and ‘sec2’ (**Supplementary Table 2**). In addition, 1-mismatch epitopes were considered and characterized in the alpha-gliadin amplicon analysis. The abundance of CD epitopes was calculated for each genotype according to the abundance of the amplicons containing them. We have also classified the alpha- and gamma-gliadin amplicons into ‘types’ considering the total number of DQ epitopes present in them, e.g. the Alpha_0 ‘type’ comprises all the alpha-gliadin amplicons with no epitopes, while the Alpha_7 amplicons contain 7 epitopes. For the alpha-gliadin amplicon, the p31-43 peptide was analyzed independently, and it was not included in the amplicons ‘types’ classification. The abundance of amplicon ‘types’ was also calculated as described previously.

### 2.4 Ranking of cereal genotypes’ immunogenic potential by analytic hierarchy process

In order to study the immunogenic potential of the genotypes assayed in the present work, we got a synthetic value (score) to rank the genotypes crossing their epitope abundance with a previous immunogenicity screening of prolamin peptides encompassing CD epitopes (Tye-Din et al., 2010). We searched for the known CD epitopes in the list of oligopeptides and crossed their scores with our values of epitope abundance in each genotype based on the analytic hierarchy process (AHP) method (Saaty, 2008) implemented with AHPy library. We added the abundance data from the amplicons ‘types’ to the process, getting a scheme with one criterion of first level and two criteria of second level (**Figure 2**). The complete methods were described on https://github.com/MiriamMarinS/wheatAHP. To calculate the final score for the alternative genotypes (**Figure 2**), the log_2_(FC) of the abundance was calculated for each pair-wise comparison between each pair of genotypes, and this measure was transformed into intensity values according to the AHP method. In the first criterion comprising the epitopes vs amplicons ‘types’ comparison, we gave equal or more weight to the first one in order to highlight the epitope sequence rather than the number of copies across the amplicon. Once the intensity values favoring the amplicons ‘types’ were removed, the remaining nine possible values were used for this criterion, giving nine immunogenic potential scores for each genotype. From this, the mean and the standard error were used in the ranking representation. The source code is available at https://github.com/MiriamMarinS/wheatAHP.

### 2.5 Statistical analysis

The t-test analyzes were performed using R software (Core Development Team, 2020). For the Principal Component analysis (PCA), FactoMineR (Lê et al., 2008) and Factoextra (Kassambara and Mundt, 2017) libraries were used. The unsupervised hierarchical clustering was carried out with R software in order to obtain the correlation-based distances. The heatmaps were represented using pheatmap library (Kolde and Kolde, 2018).

## 3 Results

A summary of the alpha and gamma-gliadin amplicons is presented in **Table 1**. The abundance of each amplicon for each genotype is provided in **Supplementary Tables 3, 4**. As shown, for the set of genotypes analyzed, we have found a higher number of different amplicons for the alpha- than for the gamma-gliadins. Stop codons were found in 21 of 76 alpha-gliadin amplicons and in 9 of 41 gamma-gliadin ones (**Table 1**). All the epitopes listed in Sollid et al. (2020) were searched in the translated amplicon sequences, and 17 non-deamidated ones were found. The number of CD epitopes found in the same amplicon varied between 0 and 7 for the alpha-gliadins, and between 0 and 14 for the gamma-gliadins/secalins (**Table 1**). Interestingly, 39% of the alpha-gliadin amplicons do not contain CD epitopes while only 15% of the gamma-gliadin/secalin ones were free of them. The total number of amplicons per species, and their distribution in putative genes and pseudogenes are in **Supplementary Table 5**. It is remarkable that bread wheat genotypes not containing the 1BL/1RS translocation showed a higher average number of both alpha- and gamma-gliadin epitopes than those containing such translocation.

**Table 1:** General description of the alpha and gamma-gliadin amplicons in the set of wheat and tritordeum genotypes used in this study.

Features	Alpha-gliadin	Gamma-gliadin
Total number of different amplicons	76	41^a^
Putative genes. Amplicons with no-stop codons	55	32^b^
Pseudogenes. Amplicons with stop codons	21	9
Range of amplicon length (bp)	201 - 279	216 - 507
Range of CD epitopes per amplicon. Only putative genes	0 - 7	0 - 14
Number of amplicons not containing CD epitopes (putative genes)	31 (25)	6 (3)^c^

a 7 of them are secalins.

b 4 of them are secalins.

c all of them are secalins.

### 3.1 Epitope abundance in the set of cereal genotypes

**Figure 3:**
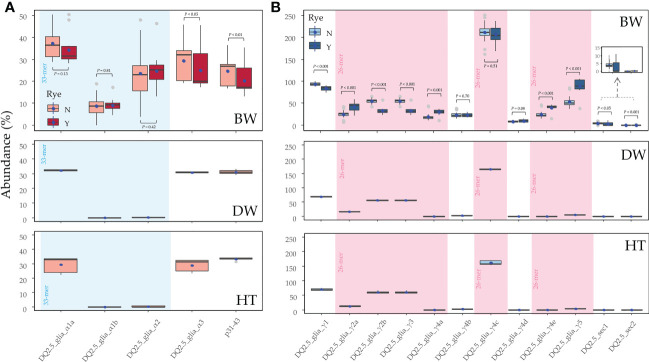
The abundance of CD epitopes in the **(A)** alpha- and **(B)** gamma-gliadin amplicons of putative genes. For bread wheat genotypes, the presence **(Y)** or not **(N)** of the rye translocation is indicated. The blue dots indicate the mean, the grey dots indicate the outliers, and the black horizontal line represents the median value. The statistical analysis for each epitope and the immunogenic peptide between the genotypes containing **(Y)** or not **(N)** the rye translocation was performed by the non-parametric Mann-Whitney-Wilcoxon test, and by the t-test for the DQ2.5_glia_α1b, DQ2.5_glia_γ1, DQ2.5_glia_γ2a, DQ2.5_glia_γ4c and DQ2.5_glia_γ4d epitopes that were normally distributed across the genotypes. The epitopes contained in the 33-mer and 26-mer regions are shaded. BW, bread wheat; DW, durum wheat; HT, tritordeum.

Next, the abundance of the CD epitopes in the three species was calculated considering the abundance of the amplicons which carry them in each genotype (**Supplementary Tables 6, 7**). The list of epitopes/peptides found comprises the p31-43 and four DQ2.5 epitopes for the alpha-gliadin amplicons; and 12 DQ2.5 epitopes, including two secalin ones, for the gamma-gliadin/secalin amplicons. Although the bread wheat genotypes showed the highest abundance of epitopes, they also had wider heterogenicity on their abundance profiles than the other cereals (**Figure 3**). In contrast, durum wheat genotypes had the lowest number of epitope matches.

In the alpha-gliadin amplicons, there were marked differences between the three cereal species for the abundance of the CD immunogenic epitopes. In the bread wheat genotypes, the DQ2.5_glia_α1a was the highest abundant among the three epitopes included in the 33-mer peptide (**Figure 3A**) while DQ2.5_glia_α1b was the less one. If bread wheat with and without the rye translocation were compared, the significant effects in the alpha-gliadin epitopes were shown, involving a less abundance of DQ2.5_glia_α3 epitope and p31-43 peptide (**Figure 3A**). In contrast to bread wheat, durum wheat and tritordeum genotypes had null or closer to zero abundance of the DQ2.5_glia_α1b and DQ2.5_glia_α2 epitopes (**Figure 3A**).

In the case of the gamma-gliadin/secalin amplicons, the DQ2.5_glia_γ4c epitope, included in the 26-mer peptide, stood up in all three cereal species (**Figure 3B**). In addition, the DQ2.5_glia_γ1, DQ2.5_glia_γ2b, and DQ2.5_glia_γ3 epitopes had also high abundance in all the cereal crops studied. The last two epitopes had the same abundance because they were present in the same gamma-gliadin amplicons (**Supplementary Table 4**). The rest of the epitopes were poorly represented in durum wheat and tritordeum genotypes, while for bread wheat, other epitopes such as the DQ2.5_glia_γ5, included in the 26-mer peptide, had also a high abundance (**Figure 3B**). Comparing the abundance of bread wheat genotypes with and without the rye translocation, the most abundant epitope DQ2.5_glia_γ4c was equally represented in both. Interestingly, the epitopes DQ2.5_sec1 can also be found in the gamma-gliadins amplicons (**Figure 1B**), in fact, there were no differences in the abundance of this epitope between bread wheat with and without the rye translocation (**Figure 3B**). On the other hand, the abundance of the DQ2.5_sec2 epitope was significantly higher in the genotypes containing the rye translocation (**Figure 3B**), since this epitope was only present in one 40-k-γ-secalin (**Supplementary Table 4**), Moreover, epitopes DQ2.5_glia_γ4b and DQ2.5_glia_γ4c were also present in rye secalins.

### 3.2 Gliadin amplicons are grouped into different types depending on the number of CD epitopes

**Figure 4:**
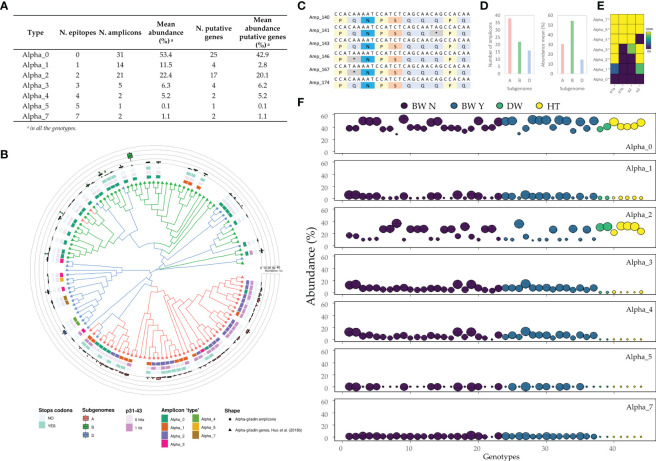
Characterization of the alpha-gliadin’s amplicons ‘types’. **(A)** The abundance of each alpha-gliadin ‘type’. **(B)** Phylogenetic tree using the maximum-likelihood method in MEGA 10.1.7 from a MUSCLE sequence alignment (default parameters). From outside to inside: (i) the abundance for all the genotypes of each amplicon ‘type’, (ii) presence of premature stop codons, (iii) hits of the p31-43 peptide, and (iv) the number of DQ2.5 epitopes in their translated peptide sequence. **(C)** Stop codons generated by the C T mutation. **(D)** Number and mean abundance (%) per subgenome. **(E)** Frequency with which epitope appears in each amplicon ‘type’: the number of amplicons containing each epitope divided by the total amplicons classified in this ‘type’. **(F)** The abundance of each alpha-gliadin’s amplicon ‘type’ for each wheat and tritordeum genotype based on putative genes. BW N: bread wheat without rye translocation; BW Y: bread wheat with rye translocation; DW: durum wheat; HT: tritordeum. The dot sizes ranged from the lowest (smallest) to the highest (biggest) abundance of all genotypes within each amplicon ‘type’.

**Figure 5:**
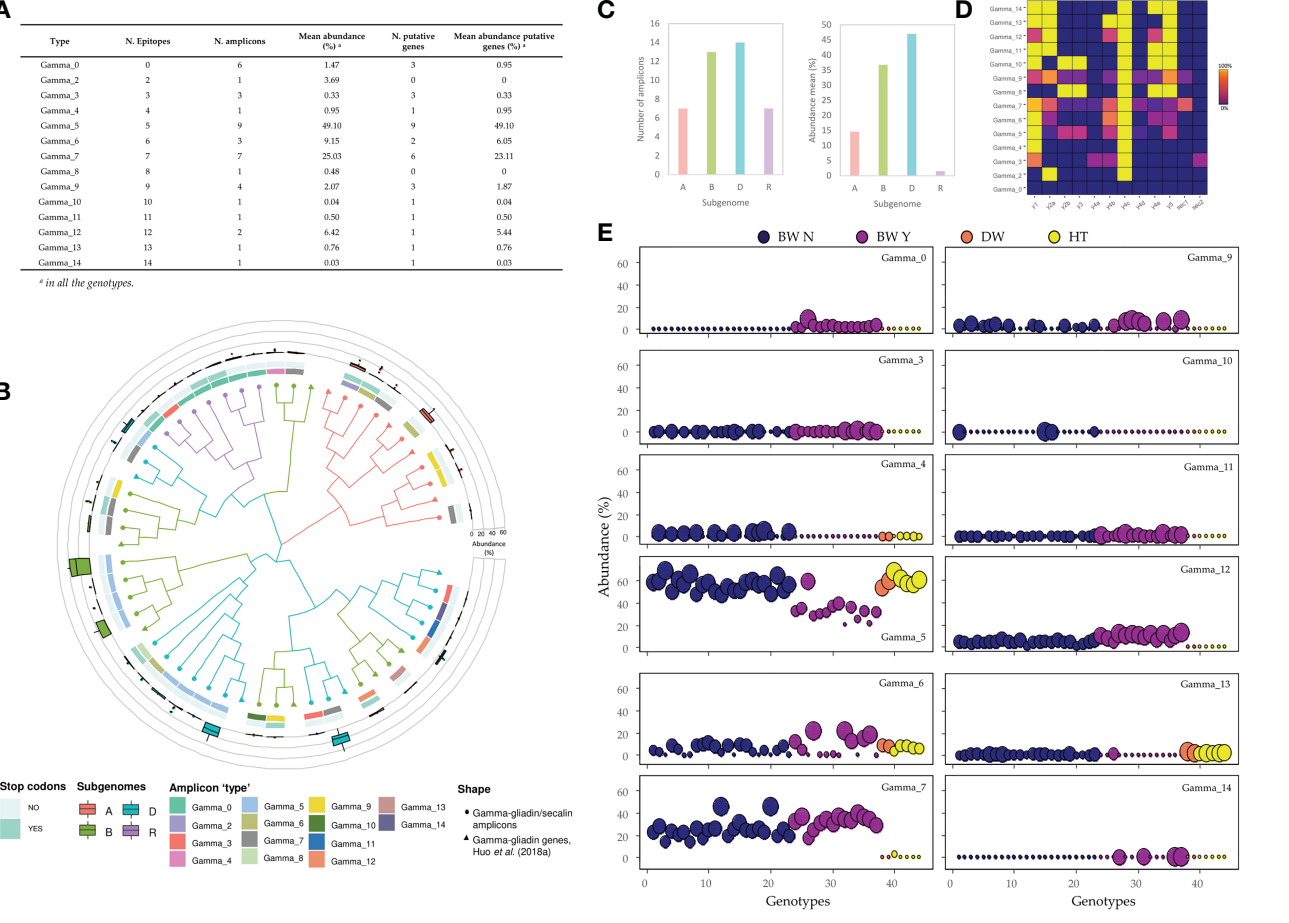
Characterization of the gamma-gliadin’s amplicons ‘types’ **(A)** The abundance of each gamma-gliadin ‘type’. **(B)** Phylogenetic tree using the maximum-likelihood method in MEGA 10.1.7 from a MUSCLE sequence alignment (default parameters). The amplicons were assigned to each subgenome by their similarity with the gamma-gliadin genes identified in Huo et al. (2018a) for the Chinese Spring. From outside to inside: (i) the abundance for all the genotypes of each amplicon ‘type’, (ii) presence of premature stop codons, (iii) hits of the p31-43 peptide, and (iv) the number of DQ2.5 epitopes in their translated peptide sequence. **(C)** Number and mean abundance (%) per subgenome. **(D)** Frequency with which epitope appears in each amplicon ‘type’: the number of amplicons containing each epitope divided by the total amplicons classified in this ‘type’. **(E)** The abundance of each gamma-gliadin’s amplicon ‘type’ for each wheat and tritordeum genotype based on putative gene amplicons. BW N: bread wheat without rye translocation; BW Y: bread wheat with rye translocation; DW: durum wheat; HT: tritordeum. The dot sizes are ranged from the lowest (smallest) to the highest (biggest) abundance of all genotypes within each amplicon ‘type’.

The 33-mer is one of the most immunogenic peptides described for the alpha-gliadins, and it is related to the presence of six overlapping copies of three CD epitopes (Shan et al., 2002). However, the 33-mer region showed a high sequence heterogeneity in the alpha-gliadin amplicons and not all of them presented the six epitopes expanding the 33-mer (**Figure 1A**). This is clear for the durum wheat and tritordeum genotypes, which do not contain the full 33-mer and only have 2 of the 3 epitopes comprising the complete immunogenic peptide. Similarly, the gamma-gliadin amplicons presented regions that comprise many overlapping epitopes with a high sequence variability (**Figure 1B**). The total amplicons for both the alpha and gamma-gliadins/secalins differed in the number of CD epitopes in their peptide sequences, comprising a range of 0 - 7 and 0 - 14 epitopes respectively (**Table 1**). Based on these differences, we were able to group the alpha and gamma-gliadin/secalin amplicons into 7 and 14 ‘types’, respectively, named accordingly as Alpha_0 to Alpha_7 and Gamma_0 to Gamma_14 (**Figures 4**, **5**). In addition, their abundance was calculated for each genotype and summarized in **Supplementary Tables 8, 9**.

As shown, the Alpha_0 - not containing CD epitopes - stood out as the alpha-gliadin amplicon ‘type’ with the highest overall abundance (53.4%), being comprised mainly of putative genes, followed by the Alpha_2 and Alpha_1 ‘types’ (**Figure 4A**). On the other hand, only two alpha-gliadin amplicons contained seven CD epitopes (Alpha_7) with an abundance of 1.1% in all the genotypes (**Figure 4A**). The Alpha_7 amplicons presented the six overlapping epitopes comprising the 33-mer and the DQ2.5_glia_α3 downstream epitope (**Figure 4E**), showing the same epitope distribution represented in **Figure 1A**. Next, we assigned each amplicon to the A, B, and D-subgenomes by finding specific motifs in their translated peptide sequence as described in van Herpen et al. (2006). In addition, the Chinese Spring (CS) alpha-gliadin genes (Huo et al., 2018b) were also included in the phylogenetic tree as references. However, not all the sequences in a cluster were assigned to the same subgenome, i.e. there was no homogeneity in the subgenome assignation despite the sequence similarity of the elements in the cluster (**Figure 4B**). The most abundant alpha-gliadin amplicon in almost all the genotypes from the three genotypes belonged to the B-subgenome (**Figures 4B, D**). This amplicon, and most of the B-subgenome ones - except three of them which contain one epitope - were classified as Alpha_0 ‘type’. In addition, only one B-subgenome amplicon had the p31-43 peptide, but this peptide was mainly overrepresented in the A-subgenome amplicons (**Figure 4B**). On the other hand, amplicons with a high number of epitopes, including the Alpha_7, belong to the D-subgenome (**Figure 4B**). The Olaeta Artillero and ACA 321 genotypes showed the highest abundance for the Alpha_7 ‘type’, being both bread wheat without the rye translocation (**Figure 4F**, **Supplementary Table 8**). Interestingly, the abundance of the Alpha_3 to Alpha_7 ‘types’ was very low or even absent for the durum wheat and tritordeum genotypes. In contrast, the Alpha_2 stood out for these two crop species and comprised amplicons harboring the DQ2.5_glia_α1a and DQ2.5_glia_α3 epitopes (**Figures 4F, E**). About 27% of the alpha-gliadin amplicons presented premature stop codons and were considered pseudogenes (**Table 1**). Some of them proceed from the mutation of C → T in the glutamine codons (**Figure 4C**). These pseudogene amplicons were mainly presented in the A-subgenome as well, followed by the B-subgenome (**Figure 4B**).

Regarding the gamma-gliadins/secalins analysis, there were only six amplicons without CD epitopes with a low mean abundance of 1.47%, being only present in bread wheat genotypes with rye translocation as these amplicons were the 40k-γ-secalins (**Figure 5A**). However, these genotypes presented also the Gamma_14 ‘type’, which contained the highest number of epitopes matches in the set of amplicons ‘types’. This ‘type’ consisted of five DQ2.5 gamma-gliadin epitopes repeated across the amplicon sequences and had a very low abundance (**Figures 5B, D**; **Supplementary Table 9**). The most abundant gamma-gliadin ‘types’ were Gamma_5 and Gamma_7, with a high variability of DQ2.5 epitopes combinations in their amplicon sequences, and comprising 9 and 6 putative genes respectively (**Figures 5A, D**). The first one was found in all the genotypes, including bread and durum wheat, and tritordeum genotypes, however, their abundances were slightly lower for many of the bread wheat with the rye translocation (**Figure 5E**). In the case of Gamma_7, it was only present in bread wheat, except for the tritordeum genotype Aucan, being proINTA Gaucho – not containing the rye translocation – the genotype with the highest abundance for this gamma-gliadin amplicon ‘type’ (**Supplementary Table 9**; **Figure 5E**). As for the alpha-gliadins, the gamma-gliadin amplicons were assigned to subgenomes, using the CS genes as references. The most abundant amplicons were from the D-subgenome, followed by B and A ones (**Figures 5B, C**). Interestingly, the Gamma_14 was only present in the D-subgenome, and the Gamma_0 was found only in the secalin amplicons, as stated before (**Figure 5B**). The pseudogenes were found in all three subgenomes, with a slightly higher proportion in the B one and secalin amplicons (**Figure 5B**). The Gamma_10, Gamma_13 and Gamma_4, only found in the B-subgenome, were absent in bread wheat genotypes with rye translocation. As an exception, the Gamma_13 presented only a low abundance in BIONTA 2004, which had not the complete *Gli-B1l* allele in the A-PAGE profile (**Supplementary Table 9**; **Supplementary Figure 1**).

### 3.3 Clustering of wheat and tritordeum genotypes based on CD epitopes profile

**Figure 6:**
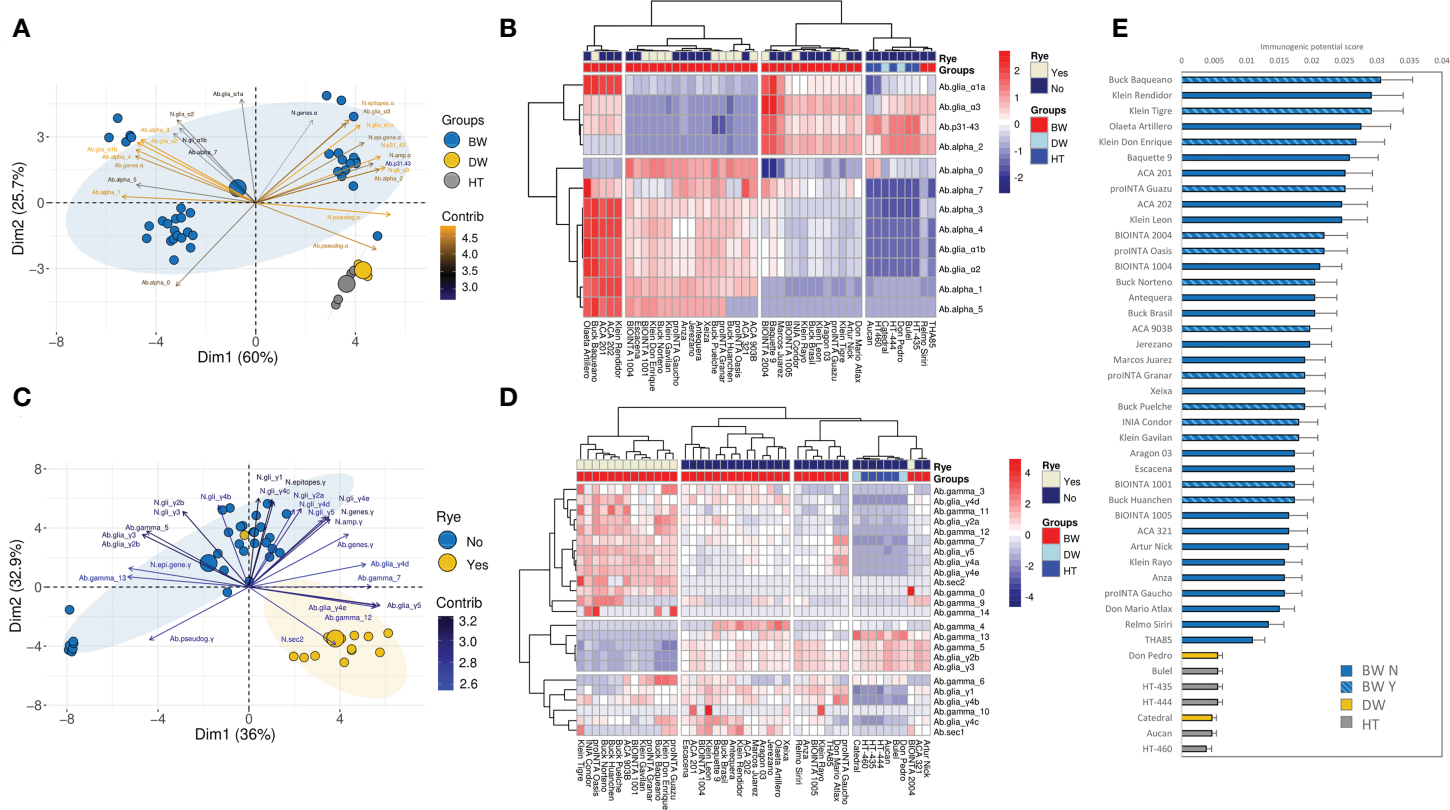
Principal Component Analysis (PCA) for the **(A)** alpha- and **(C)** gamma-gliadin amplicons. The contribution of each variable to the variance of the model is indicated by a color code. The big size dots indicate the center of the ellipses with 95% of confidence per each group of genotypes. Because of the high number of variables for the gamma-gliadin amplicon, the top 25 variables that most contribute to the variance of the model were included to improve the visualization of the results. Heatmap of the scaled abundance of the CD epitopes and the amplicons ‘types’ for alpha- **(B)** and gamma-gliadin **(D)** amplicons. The dendrogram based on Pearson and Spearman correlation distance, for alpha- and gamma-gliadin amplicons respectively, is represented for the epitopes and amplicons ‘types’, and for the bread wheat (BW), durum wheat (DW), and tritordeum (HT) genotypes. The rye translocation is indicated with Yes/No in the Rye legend. **(E)** Ranking of genotypes based on their immunogenic potential score. The bars indicate the mean plus the standard error.

A PCA and unsupervised hierarchical clustering were carried out for the alpha- and gamma-gliadin/secalin amplicons separately, comprising variables such as the abundance of the CD epitopes, the p31-43 peptide, and the amplicons ‘types’. Other parameters, like the number of CD epitopes and the number of putative genes and pseudogenes, were included in the PCA (**Figure 6**).

The genotypes assayed were separated into four clusters based on the alpha-gliadin amplicon results (**Figure 6B**). One of the clusters comprised the durum wheat and tritordeum genotypes. Interestingly, two bread wheat genotypes – Relmo Siriri and THA85 – were in this cluster (**Figure 6B**). The main features of genotypes from this cluster were a lower abundance for all the CD epitopes and alpha-gliadin amplicons ‘types’, but no for the p31-43 peptide, the Alpha_2, and the DQ2.5_glia_α3. The other three clusters were comprised of bread wheat genotypes and were clearly separated in the PCA analysis (**Figures 6A, B**). The second and third ones - from left to right side in **Figure 6B** - had an opposite abundance profile concerning epitopes like the DQ2.5_glia_α1a, DQ2.5_glia_α3, the p31-43 peptide, and the Alpha_2 amplicon ‘type’, which were abundant in the third cluster and poorly represented in the second one. In contrast, the Alpha_0 was less abundant in the third cluster than in the second one (**Figure 6B**). The remaining five bread wheat genotypes were clustered together and presented a high abundance for all de the CD epitopes and amplicon ‘types’, except for the Alpha_2, and Alpha_0 (**Figure 6B**). Olaeta Artillero and ACA 321 stood out because of their high abundance of Alpha_7, which contains the full 33-mer peptide (**Figure 6B**).

Regarding the gamma-gliadin/secalin amplicon, the hierarchical clustering provided four clusters (**Figure 6D**). The durum wheat and tritordeum genotypes were grouped together in one of them and presented lower abundance for almost all the CD epitopes and gamma-gliadin amplicon ‘types’ (**Figure 6D**). Interestingly, the bread wheat genotypes were well separated by the presence of rye translocation, and only BIOINTA 2004 (containing the rye translocation) was grouped with the bread wheat genotypes without secalins (**Figures 6C, D**). In general, bread wheat genotypes grouped in the second and third clusters - from left to right side in **Figure 6D** - had a high variability of profiles. However, the first cluster located on the far left, comprising bread wheat genotypes with the rye translocation, had a contrasting profile than the durum wheat and tritordeum genotypes, particularly, the Gamma_0 amplicon ‘type’ was higher abundant in this cluster than in the rest of them, as for the DQ2.5_sec2 epitope and Gamma_14 in some of the genotypes (**Figure 6D**).

The linkage of the abundance of the epitopes and the amplicons ‘type’ of all the genotypes with previous immunogenic screening of oligopeptides encompassing CD epitopes, gave as a result the ranking of the genotypes based on their immunogenic potential score (**Figure 6E**). As it is shown, the durum wheat-based genotypes had the lower scores, far away from the first-positioned bread wheat genotypes (**Figure 6D**). Comparing this ranking with the genotypes clusterization in **Figures 6B, D**, Olaeta Artillero was maintained as one of the most potential immunogenic genotypes as it was present among the first places in the ranking, while ACA 321 had a much lower score despite having a 33-mer abundance similar to Olaeta Artillero. This was due to ACA 321 presenting low overall epitope abundance for the gamma-gliadin amplicons, close to the durum wheat-based genotypes (**Figure 6E**). Many of the first-ranked genotypes had a high content of alpha-gliadin epitopes and the 33-mer peptide, and a low abundance of the Alpha_0 ‘type’ (**Figure 6E**). Interestingly, the presence of the rye translocation did not seem to have a significant role in this ranking, as all the bread wheat genotypes with secalins were intercalated with the others (**Figure 6E**).

### 3.4 Identification of CD alpha-gliadin epitopes with one mismatch

**Figure 7:**
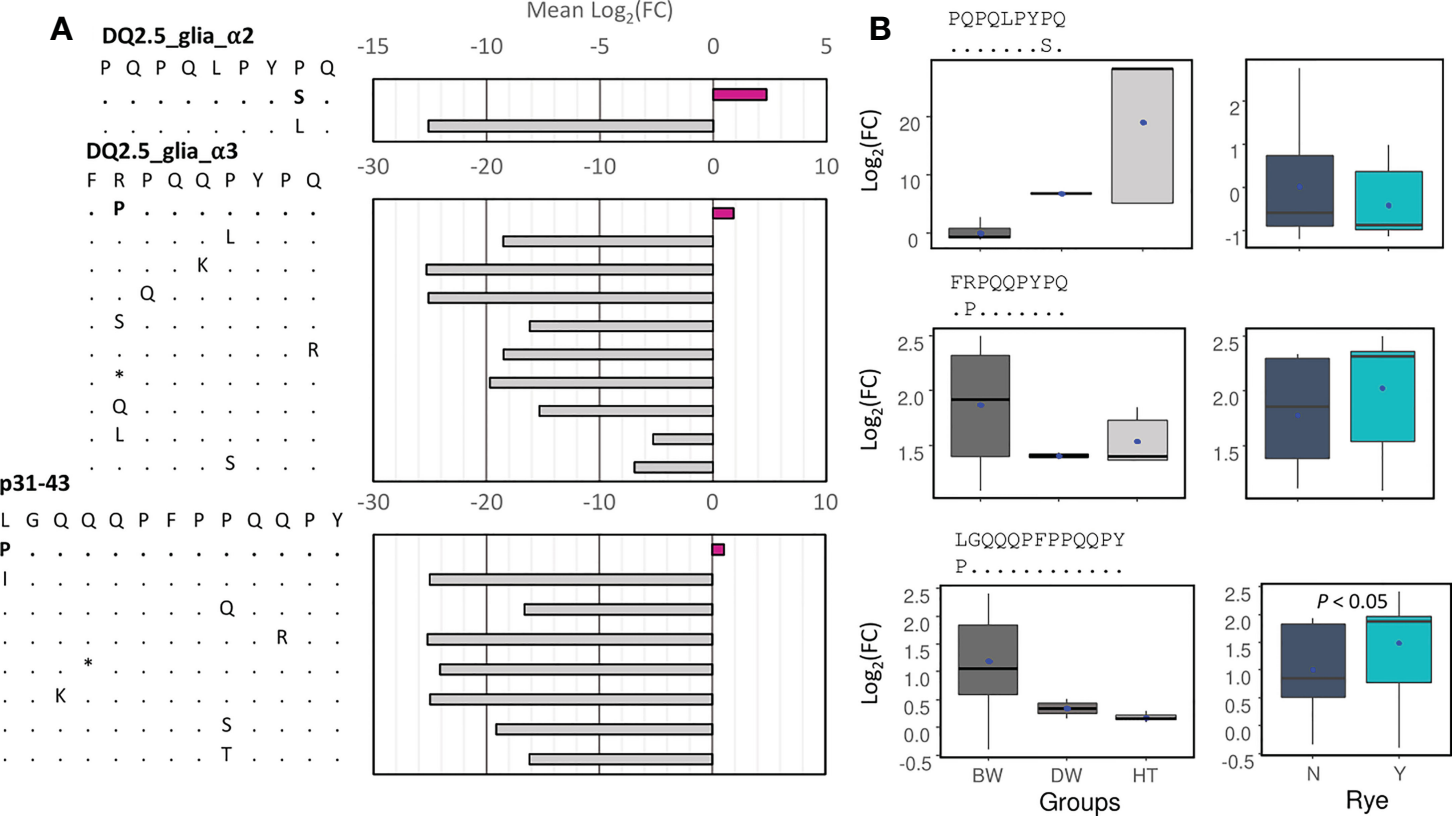
Analysis of epitopes’ variants (one mismatch). **(A)** Mean of the log_2_(FC) between the abundance of the epitopes’ variants and the canonical epitopes, or the immunogenic peptide in the case of p31-43. **(B)** Boxplots for the three epitopes’ variants with the log_2_(FC) > 0 for wheat and tritordeum genotypes (left) and for the presence of the rye translocation only in bread wheat genotypes (right). The log_2_(FC) was calculated as follows: the abundance of the epitope’ variant/abundance of the canonical epitope. For the rye boxplots, the Mann-Whitney-Wilcoxon test was performed, and only the significant *P*-value was indicated. The blue dots indicate the mean value.

The CD alpha-gliadin epitopes containing one mismatch with respect to the canonical ones were identified, and their abundance was also calculated (**Figure 7**). Next, the log_2_(FC) between them and their canonical epitopes was measured (**Supplementary Table 10**). Only for three of them, the log_2_(FC) mean was higher than zero, which means that these epitope variants were more abundant than the canonical ones in most of the genotypes (**Figure 7A**). The S variant of DQ2.5_glia_α2 (PQPQLPY**S**Q) had higher abundance in durum wheat and tritordeum genotypes, while the P variant of DQ2.5_glia_α3 and the p31-43 P variant were more abundant in bread wheat genotypes, being the last one overrepresented in the genotypes containing the rye translocation (**Figure 7B**).

## 4 Discussion

RNAi technology has been used to down-regulate the genes encoding for the gliadins, the major responsible for triggering CD. As a result, a reduction of up to 98% for these proteins was reported in some wheat genotypes (Gil-Humanes et al., 2010). More recently, CRISPR gene editing was used to knock out the alpha-gliadins multigene family (Sánchez-León et al., 2018). The use of these techniques is not without controversy; while some countries have stated that foods derived from edited crops using CRISPR technology do not require prior approval if they meet certain safety criteria and do not contain genetic material from foreign organisms, in other countries, particularly in the European Union, gene-edited foods are subject to very strong regulation (European Union Novel Foods Regulation, EC 258-97), which greatly limits the development and marketing of such products. In this context, the identification of wheat varieties with low content of immunogenic epitopes related to CD is still an important objective to develop final food products or to use them as parents in future breeding programs, either by classical breeding or by biotechnological tools. In particular, for the use of gene editing techniques, it is essential to use wheat varieties with low gliadin structural complexity, either with a low number of genes or with a low number of epitopes. However, *in vitro* culture is an important bottleneck in the application of CRISPR/Cas to elite ‘cultivars’ for developing new genotypes. Recent studies reported the use of embryogenesis-related transcription factors such as *BABY BOOM (Bbm)*, *GROWTH-REGULATING FACTORs (GRFs)* or *Wuschel-related homeobox 5 (WOX5)*, which improve embryogenesis, tissue culture regeneration and plant transformation efficiency, overcoming the genotype dependency for wheat genetic transformation (Bilichak et al., 2018; Debernardi et al., 2020; Wang et al., 2022), and expanding the application of genome-editing to elite wheat ‘cultivars’.

Diverse approaches involving long-read sequencing, RNA and DNA amplicon sequencing, and proteomic analysis have been used to study the CD epitopes in the gliadin sequences of wheat. Most of them were focused on the potential immunogenicity of alpha-gliadins in *Triticum turgidum, Triticum aestivum* and their ancestors, being studied to look for the T cell stimulatory CD epitopes (Salentijn et al., 2013; Ozuna et al., 2015; Huo et al., 2018b; Halstead-Nussloch et al., 2021; Schaart et al., 2021). However, there are fewer studies involving other gliadin groups such as the gamma-gliadins in terms of the presence and abundance in CD epitopes (Wang et al., 2017; Cho et al., 2018). In this work, we have analyzed the immunogenic gliadin complexes located on the repetitive domain of the alpha- and gamma-gliadins in a set of bread and durum wheat, and tritordeum genotypes by high throughput amplicon sequencing. The two amplicon databases obtained were compared to the gene sequences of the alpha- and gamma-gliadins published for the CS (Huo et al., 2018a; Huo et al., 2018b). Assignment to the three wheat subgenomes of the gamma-gliadin amplicons by their proximity to the gene sequence from CS in the phylogenetic tree resulted in a total of 7, 13, and 14 amplicons assigned to A-, B- and D-subgenomes respectively. In addition, seven 40k-γ-secalin amplicons were identified, as they are located in the short arm of the 1R chromosome of rye (Kozub et al., 2014), present on the bread wheat genotypes with the 1BL/1RS rye translocation. However, the subgenome assignation of alpha-gliadin amplicons either by searching subgenome-specific motifs around the 33-mer region (van Herpen et al., 2006) or by their similarity to CS reference genes (Huo et al., 2018b) had discrepancies. Actually, the motifs employed for subgenome assignation were searched in the CS reference genes, aligning themselves in genes from different subgenomes to that of the motif itself. However, most of the A-subgenome amplicons by alpha-gliadin motifs were clustered together, as were many of the D- and B-subgenomes.

Both the alpha- and gamma-gliadins presented a high rate of pseudogenes due to the high percentage of glutamine residues encoded by CAA and CAG codons that can be mutated into stop codons through C T transition (Huo et al., 2018b; Paris et al., 2021). We report 27.6% and 22.0% of pseudogenes for the alpha- and gamma-gliadin amplicons, and many of them presented the mutation of the glutamine into a stop codon, mainly in the alpha-gliadins. However, the number of pseudogenes could be underestimated because the amplicon does not cover the entire sequence of both gliadins genes as reported by Sánchez-León et al. (2021). In fact, there was previously described 45% of pseudogenes in alpha-gliadins, while the gamma-gliadin-genes presented 21% of them, close to our results for this family (Huo et al., 2018a; Huo et al., 2018b). The alpha-gliadin amplicons that presented premature stop codons in the first repetitive domain could be regulated by a mechanism called nonsense-mediated mRNA decay (NMD) at the post-translational level so that the production of truncated proteins is prevented (Hug et al., 2016). We have shown that pseudogenes were strongly ligated to the subgenomes A and B, mainly for the alpha-gliadins. The high rate of pseudogenes detected in durum wheat and tritordeum could contribute to explaining the lower immunogenicity reported for these genotypes (Auricchio et al., 1982; Vaquero et al., 2018). However, both durum wheat and tritordeum are cereals containing gluten and therefore not suitable for celiac patients. Recently, Vriz et al. (2021) proposed a ranking of CD epitopes based on their enzymatic degradation, HLA-DQ binding affinity, and T cell activation, including the data from Tye-Din et al. (2010), which allows for determining their potential to trigger CD. In this ranking, the alpha-gliadin epitopes were the most immunodominant. However, there was an exception for the DQ2.5_glia_α3 epitope because of its chymotrypsin degradation decreasing its adequate binding to HLA-DQ receptors. Interestingly, for the set of genotypes analyzed in this work, about 40% of the alpha-gliadin amplicons which an overall abundance of 53.4%, mainly located in the B-subgenome, had no CD epitopes. This is in agreement with previous works which reported that alpha-gliadin genes containing few CD epitopes or a few copies of them were in the B-subgenome (Wang et al., 2017; Halstead-Nussloch et al., 2021). Among the amplicons with CD epitopes, there were two alpha-gliadin epitopes with a low or null abundance in durum wheat and tritordeum. In those genotypes, the epitope with a high abundance was DQ2.5_glia_α3, which is the less immunogenic alpha DQ2.5 epitope as mentioned before (Anderson et al., 2006). The fact that the durum wheat and tritordeum genotypes lack two of the epitopes included in the 33-mer implies that this peptide cannot be found in these genotypes, and therefore they also lack the Alpha_7 amplicon ‘type’, which comprises the six overlapping epitopes of the 33-mer and one DQ2.5_glia_α3 downstream. The 33-mer was only present in alpha-gliadin amplicons from the D-subgenome, and at low frequency, which means that there are only few copies of the gene coding this peptide. The low frequency of the 33-mer in bread wheat was reported previously (Ozuna et al., 2015; Huo et al., 2018b). The same works proposed that the origin of the 33-mer was probably due to variation events of *Aegilops tauschii* alpha-gliadin genes, supporting the fact that this immunogenic peptide can only be found on the D-subgenome.

In addition to the 33-mer, the p31-43 peptide was reported to activate the innate immune response inducing a non-HLA-mediated inflammatory reaction. Its immunogenicity was proved in both *in vitro* and *in vivo* studies (Maiuri et al., 1996; Gianfrani et al., 2005; Barone et al., 2014). This peptide was mainly present in the alpha-gliadin amplicons from the A-subgenome, in agreement with the work of Halstead-Nussloch et al. (2021). This peptide had a conserved high abundance in most of the genotypes compared to some of the alpha-gliadin DQ2.5 epitopes. In addition, the p31-43P epitope variant, which has been described as an immunogenic peptide for CD patients (Maiuri et al., 1996; Maiuri et al., 2003), was more represented overall than the canonical peptide in bread wheat, especially for the genotypes containing the rye translocation. There were also other epitope variants more abundant than the canonical epitopes. For example, the DQ2.5_glia_α3, whose T cell stimulatory capacity was destroyed for the FPPQQPYPQ (R to P substitution) epitope variant (Mitea et al., 2010), and which was mostly represented in bread wheat. Furthermore, the DQ2.5_glia_α2 PQPQLPYSQ variant (P to S substitution) showed comparable T-cell proliferation and anti-33-mer binding capacity than its canonical CD epitopes (Ruiz-Carnicer et al., 2019), one of the major epitopes recognized by most of CD patients (Tye-Din et al., 2010). However, this epitope variant showed also 1,000 times reduced DQ2.5_glia_α2 specific T cell stimulation compared to the canonical epitope in a previous work (Mitea et al., 2010). Interestingly, this variant was more abundant on tritordeum genotypes and was found only in the A-subgenome (Ruiz-Carnicer et al., 2019). Overall, there is a strong relationship between the CD epitope subgenomes and their immunogenicity, those with the highest immunogenic potential the ones from the A- and, mainly, the D-subgenome (Halstead-Nussloch et al., 2021). Thus, durum wheat and tritordeum genotypes would have a lower immune response in CD patients and fewer gluten immunogenic peptides (GIPs) are expected in the excretion of healthy individuals (Auricchio et al., 1982; Vaquero et al., 2018). However, there was a high heterogeneity of the alpha-gliadin amplicon abundance profile from the three subgenomes in bread wheat, so there are genotypes with a low-immunogenic potential as they have clustered together with the durum wheat and tritordeum: Relmo Siriri and THA85, for example. Actually, THA85 is the only bread wheat genotype in which we have not found the complete 33-mer peptide. Although this will have to be corroborated in subsequent analyses, it is ranked as one of the best candidates for precision breeding programs in order to obtain low-immunogenic wheat. This clusterization also allowed us to identify five bread wheat with the highest immunogenic potential based on the alpha-gliadin amplicon results, including only one with the rye translocation. Clearly these genotypes should be avoided in breeding programs to obtain varieties with low immunogenicity.

As mentioned before, there are very few studies that have simultaneously categorized the CD epitope content in both the alpha- and gamma-gliadins in wheat genotypes. Actually, the gamma-gliadin genes have more diverse and numerous CD epitopes, as described in Wang et al. (2017). A total of 10 gamma-gliadin HLA-DQ2.5 epitopes and two epitopes of secalins were mapped to the gamma-gliadin/secalin amplicons which include the 26-mer region. This peptide is highly resistant to intestinal brush border membrane proteolysis and, as it is multivalent, the intact 26-mer peptide is more antigenic compared to its smaller monovalent counterparts (Shan et al., 2005). Like the 33-mer, the 26-mer is only present in the D-subgenome, and at a low frequency in the transcripts of bread wheat (Salentijn et al., 2012). Of the epitopes included in this region, the most abundant was DQ2.5_glia_γ4c in all the genotypes, independently of their genetic background, as it presents a high number of copies along the gamma-gliadin amplicon. In addition to this, other epitopes also presented in the 26-mer region had a higher abundance in bread wheat, with significant differences between those with and without the rye translocation. The bread wheat genotypes with the 1BL/1RS translocation had a higher abundance of the epitope DQ2.5_glia_γ5, reported as one of the gamma-gliadin epitopes with higher immunological relevance (Vriz et al., 2021). Only one 40k-γ-secalin amplicon showed the epitope DQ2.5_sec2, in combination to DQ2.5_glia_γ4b and DQ2.5_glia_γ4c, while the rest of secalin amplicons do not contain any epitope matches. As shown in previous research, the 40k-γ-secalins present few epitope hits, including the DQ2.5_glia_γ4c between them. In fact, this secalin type has less epitope than the rest of rye prolamins such as ω-secalins or 75k-γ-secalins (Lexhaller et al., 2019), whose genes are present in the 2R chromosome (Kozub et al., 2014). The other 40k-γ-secalins found on these bread wheat genotypes comprised the Gamma_0 ‘type’, only present in wheat containing the rye translocation. However, these genotypes were also the only ones to present the gamma-gliadin amplicon with the highest number of CD epitopes (Gamma_14), which belonged to the D-subgenome. There were three amplicons ‘types’ present only in the B-subgenome. All of them were absent in the bread wheat with the 1BL/1RS translocation, as 1BS chromosome were fully or partially depleted in those genotypes. However, BIOINTA 2004 was the exception, as it had one of those amplicons ‘types’ at low abundance. The *Gli-B1l* allele, associated with this translocation, was not complete in its A-PAGE protein profile, so a partial translocation – not centromeric translocation - could explain the differences between BIOINTA 2004 and the other genotypes with the rye translocation.

The durum wheat and tritordeum genotypes had a lower abundance for most of the epitopes and amplicon ‘types’, including the Gamma_14. Like alpha-gliadins, the gamma-gliadins presented less immunogenic elements for those genotypes, which makes them good candidate for obtaining varieties with low-immunogenic gluten by precision breeding programs. This also applies to the bread wheat genotypes clustered with the durum wheat and tritordeum ones which have a low epitopes’ abundance profile, including one genotype with the rye translocation (BIOINTA 2004). The rest of the bread wheat genotypes containing the translocation were clustered together and presented a high abundance of many epitopes and amplicon ‘types’, including Gamma_0.

The clusterization of genotypes by the alpha- and gamma-gliadin amplicons allowed us to identify groups of genotypes that could be potential candidates for precision breeding programs. However, the hierarchical clustering reflects only the abundance of the set of epitopes and amplicons ‘types’, and does not consider information on how much immunogenic each epitope is as they do not contribute equally to trigger the CD response. Connecting the results of the amplicon analysis with the scores presented for the 20-mer oligopeptides in the work of Tye-Din et al. (2010), gave rise to the ranking of the genotypes assayed in this work, ordered by their immunogenic potential score. Furthermore, this score comprises the data from both immunogenic complexes, taking into account the differences in their immunoreactivity. In this ranking, the durum wheat and tritordeum genotypes were in the last positions, which means that they presented a lower immunogenic potential. Close to these, the THA85 and Relmo Siriri bread wheats also stood out for their low scores. The score was more influenced by the epitope data from alpha-gliadins, since this complex has more immunological relevance than the gamma-gliadins (Vriz et al., 2021), also reflected in the results of Tye-Din et al. (2010). In line with the alpha-gliadins immunodominance, the bread wheat genotypes with the rye translocation, despite of the full or partial depletion of the 1BS chromosome containing the gamma-gliadin loci, were intercalated in the ranking with the other bread wheat genotypes, not having the translocation a decisive role in their immunogenic potential. In this context, wheat with the 1AL/1RS rye translocation - not assayed in the present work - would present a similar classification, as the wheat genetic background would have a major implication in their immunogenic potential due to its alpha-gliadins profile. Despite this ranking being based on previous IFN-γ ELISpot assays with Peripheral Blood Mononuclear Cells (PBMCs) from CD patients, a further ELISpot assay is needed to know the immunoreactivity of the genotypes selected for future screening. In addition, this ranking could be improved with NGS data from the omega-gliadin genes, as they have an important role in the immunogenic response in CD (Vriz et al., 2021).

As shown in this work, there is a high variability of the immunogenic potential between wheat and tritordeum. The durum wheat-based genotypes stand out for their low abundance of epitopes, mainly the high immunogenic alpha-gliadin ones, and therefore they possess a great potential as candidates for precision breeding programs. However, there was a high epitope abundance heterogeneity among bread wheats, the genotypes with an epitope profile comparable to that of durum wheat and tritordeum the best to further develop low-immunogenic varieties. The rye translocation in bread wheat can be detected and quantified, playing an important role in the classification of genotypes by their gamma-gliadin amplicon profiles. Although the rye translocation provides varieties with a lower number of epitopes, the translocation did not imply a lower immunogenic potential compared to the rest of wheats. Even though all the genotypes described in this work contain gluten, there are marked differences between them, both in the number of epitopes and in the number of amplicons and their abundances, which can be used for effective variety selection towards the development of low-gluten products, or as starting material for the application of biotechnological tools such as CRISPR/Cas.

## Data availability statement

The datasets presented in this study can be found in online repositories. The names of the repository/repositories and accession number(s) can be found below: https://www.ncbi.nlm.nih.gov/, PRJNA936674.

## Author contributions

FB conceptualized the project. FB, SS-L and MM-S designed the experiment. MM-S and FB wrote the manuscript draft. MM-S, SS-L and FB performed the specific experiments and data analysis. All authors contributed to the article and approved the submitted version.
