# Supplementary material for: Unraveling the celiac disease-related immunogenic complexes in a set of wheat and tritordeum genotypes: implications for low-gluten precision breeding in cereal crops
Source: Front Plant Sci. 2023 May 11;14:1171882. doi: 10.3389/fpls.2023.1171882 (PMC10210591; doi:10.3389/fpls.2023.1171882)
Supplement: Supplementary file 1 [file DataSheet_1.zip › Supplementary materials.docx]

Supplementary Material

Unraveling the celiac disease-related immunogenic complexes in a set of wheat and tritordeum genotypes: implications for low-gluten precision breeding in cereal crops

Miriam Marín-Sanz, Francisco Barro, Susana Sánchez-León*

*** Correspondence:** Susana Sánchez-León: ssanchez@ias.csic.es

# Supplementary Figures and Tables

## Supplementary Figures


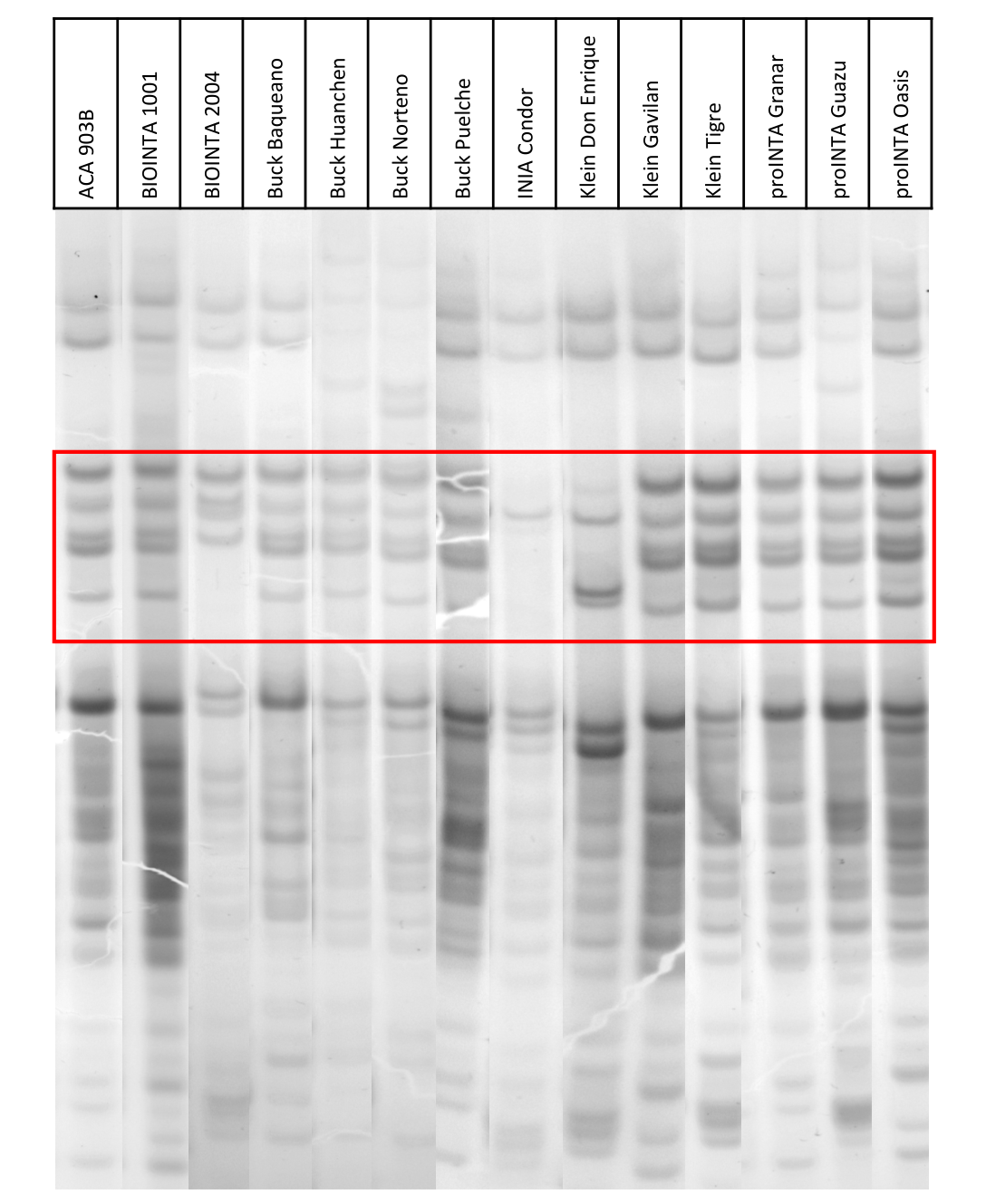


**Supplementary Figure 1.** Composition of A-PAGE gels of genotypes with rye translocation. The position of the allele *Gli-B1l* described in Metakovsky *et al.* (2018) is indicated in a red box. The original gels are in the Supplementary Figure 2.

**Supplementary Figure 2.** A-PAGE gels of genotypes assayed in the present work. The protocol followed for A-PAGE gels of total gliadin proteins is described in Gil-Humanes *et al.* (2012).

## Supplementary Tables

**Supplementary Table 1.** Genotypes of bread wheat with and without rye translocation, durum wheat and tritordeum used in the present study. BW: bread wheat, DW: durum wheat, HT: tritordeum, Y: with rye translocation, N: without rye translocation.

| **Genotype** | **Genus** | **Rye translocation** | **Provider** | **Country** | **Growth type** |
| --- | --- | --- | --- | --- | --- |
| ACA 201 | BW | N | INTA^a^ | Argentina | Spring wheat |
| ACA 202 | BW | N | INTA^a^ | Argentina | Spring wheat |
| ACA 321 | BW | N | INTA^a^ | Argentina | Spring wheat |
| ACA 903B | BW | Y | INTA^a^ | Argentina | Spring wheat |
| Antequera | BW | N | Agrovegetal | Spain | Spring wheat |
| Anza | BW | N | IAS^b^ | Spain | Spring wheat |
| Aragon 03 | BW | N | IAS^b^ | Spain | Spring wheat |
| Artur Nick | BW | N | Nickerson | Spain | Spring wheat |
| Aucan | HT | N | Vivagran S. L. | Spain | Spring wheat |
| Baquette 9 | BW | N | INTA^a^ | Argentina | Spring wheat |
| BIOINTA 1001 | BW | Y | INTA^a^ | Argentina | Spring wheat |
| BIOINTA 1004 | BW | N | INTA^a^ | Argentina | Spring wheat |
| BIOINTA 1005 | BW | N | INTA^a^ | Argentina | Spring wheat |
| BIOINTA 2004 | BW | Y | INTA^a^ | Argentina | Spring wheat |
| Buck Baqueano | BW | Y | INTA^a^ | Argentina | Spring wheat |
| Buck Brasil | BW | N | INTA^a^ | Argentina | Spring wheat |
| Buck Huanchen | BW | Y | INTA^a^ | Argentina | Spring wheat |
| Buck Norteno | BW | Y | INTA^a^ | Argentina | Spring wheat |
| Buck Puelche | BW | Y | INTA^a^ | Argentina | Spring wheat |
| Bulel | HT | N | Vivagran S. L. | Spain | Spring wheat |
| Catedral | DW | N | Eurosemillas | Spain | Spring wheat |
| Don Mario Atlax | BW | N | INTA^a^ | Argentina | Spring wheat |
| Don Pedro | DW | N | Agrovegetal | Spain | Spring wheat |
| Escacena | BW | N | Agrovegetal | Spain | Spring wheat |
| HT-435 | HT | N | Vivagran S. L. | Spain | Spring wheat |
| HT-444 | HT | N | Vivagran S. L. | Spain | Spring wheat |
| HT-460 | HT | N | Vivagran S. L. | Spain | Spring wheat |
| INIA Condor | BW | Y | INTA^a^ | Argentina | Spring wheat |
| Jerezano | BW | N | Agrovegetal | Spain | Spring wheat |
| Klein Don Enrique | BW | Y | INTA^a^ | Argentina | Spring wheat |
| Klein Gavilan | BW | Y | INTA^a^ | Argentina | Spring wheat |
| Klein Leon | BW | N | INTA^a^ | Argentina | Spring wheat |
| Klein Rayo | BW | N | INTA^a^ | Argentina | Spring wheat |
| Klein Rendidor | BW | N | INTA^a^ | Argentina | Spring wheat |
| Klein Tigre | BW | Y | INTA^a^ | Argentina | Spring wheat |
| Marcos Juarez | BW | N | INTA^a^ | Argentina | Spring wheat |
| Olaeta Artillero | BW | N | INTA^a^ | Argentina | Spring wheat |
| proINTA Gaucho | BW | N | INTA^a^ | Argentina | Spring wheat |
| proINTA Granar | BW | Y | INTA^a^ | Argentina | Spring wheat |
| proINTA Guazu | BW | Y | INTA^a^ | Argentina | Spring wheat |
| proINTA Oasis | BW | Y | INTA^a^ | Argentina | Spring wheat |
| Relmo Siriri | BW | N | INTA^a^ | Argentina | Spring wheat |
| THA85 | BW | N | Agrovegetal | Spain | Spring wheat |
| Xeixa | BW | N | IAS^b^ | Spain | Spring wheat |

^a^INTA: Instituto Nacional de Tecnología Agropecuaria

^b^IAS: Institute of Sustainable Agriculture

**Supplementary Table 2.** List of epitopes searched in the alpha-gliadin and gamma-gliadin/secalin amplicons. The epitopes were collected from the work of Sollid *et al.* (2020). The original and deamidates sequences are represented.

| **Epitope name** | **Original sequence** | **Deamidated sequence** |
| --- | --- | --- |
| DQ2.5_glia_α1a | PFPQPQLPY | PFPQPELPY |
| DQ2.5_glia_α1b | PYPQPQLPY | PYPQPELPY |
| DQ2.5_glia_α2 | PQPQLPYPQ | PQPELPYPQ |
| DQ2.5_glia_α3 | FRPQQPYPQ | FRPEQPYPQ |
| DQ2.5_glia_γ1 | PQQSFPQQQ | PQQSFPEQQ |
| DQ2.5_glia_γ2 | IQPQQPAQL | IQPEQPAQL |
| DQ2.5_glia_γ2a | FPQQPQQPF | FPEQPEQPF |
| DQ2.5_glia_γ2b | YPQQPQQPF | YPEQPEQPF |
| DQ2.5_glia_γ3 | QQPQQPYPQ | QQPEQPYPQ |
| DQ2.5_glia_γ4a | SQPQQQFPQ | SQPEQEFPQ |
| DQ2.5_glia_γ4b | PQPQQQFPQ | PQPEQEFPQ |
| DQ2.5_glia_γ4c | QQPQQPFPQ | QQPEQPFPQ |
| DQ2.5_glia_γ4d | PQPQQPFCQ | PQPEQPFCQ |
| DQ2.5_glia_γ4e | LQPQQPFPQ | LQPEQPFPQ |
| DQ2.5_glia_γ5 | QQPFPQQPQ | QQPFPEQPQ |
| DQ2.5_glia_ω1 | PFPQPQQPF | PFPQPEQPF |
| DQ2.5_glia_ω2 | PQPQQPFPW | PQPEQPFPW |
| DQ2.5_glut_L1 | PFSQQQQPV | PFSEQEQPV |
| DQ2.5_glut_L2 | FSQQQQSPF | FSQQQESPF |
| DQ2.5_hor_1 | PFPQPQQPF | PFPQPEQPF |
| DQ2.5_hor_2 | PQPQQPFPQ | PQPEQPFPQ |
| DQ2.5_hor_3a | PIPQQPQPY | PIPEQPQPY |
| DQ2.5_hor_3b | PYPQQPQPY | PYPEQPQPY |
| DQ2.5_sec1 | PFPQPQQPF | PFPQPEQPF |
| DQ2.5_sec2 | PQPQQPFPQ | PQPEQPFPQ |
| DQ2.5_sec3 | PFPQQPFQI | PFPEQPFQI |
| DQ2.5_ave_1a | PYPEQQEPF | PYPEQEEPF |
| DQ2.5_ave_1b | PYPEQQQPF | PYPEQEQPF |
| DQ2.5_ave_1c | PYPEQQQPI | PYPEQEQPI |
| DQ2.2_glut_L1 | PFSQQQQPV | PFSEQEQPV |
| DQ2.2_glia_α1 | QGSVQPQQL | EGSVQPQEL |
| DQ2.2_glia_α2 | QYSQPQQPI | QYSQPEQPI |
| DQ8_glia_α1 | QGSFQPSQQ | EGSFQPSQE |
| DQ8_glia_γ1a | QQPQQPFPQ | EQPQQPFPQ |
| DQ8_glia_γ1b | QQPQQPYPQ | EQPQQPYPE |
| DQ8_glia_γ2 | PQQSFPQQQ | PQQSFPEQE |
| DQ8_glut_H1 | QGYYPTSPQ | QGYYPTSPQ |
| DQ8.5_glia_α1 | QGSFQPSQQ | EGSFQPSQE |
| DQ8.5_glia_γ1 | PQQSFPQQQ | PQQSFPEQE |
| DQ8.5_glut_H1 | QGYYPTSPQ | QGYYPTSPQ |

**Supplementary Table 3.** Abundance of alpha-gliadin amplicons per genotype. The DNA and peptide sequence of each amplicon and the number of the CD epitopes matched are indicated.

**Supplementary Table 4.** Abundance of gamma-gliadin/secalin amplicons per genotype. The DNA and peptide sequence of each amplicon and the number of the CD epitopes matched are indicated.

**Supplementary Table 5.** The number of alpha- and gamma-gliadin/secalin amplicons, and epitopes distribution per genotype in the set of genotypes used in the study. The mean and range are indicated. Mean: average for all the genotypes classified in each specie category. Range: the minimum and maximum value found for the genotypes of each specie. Rye YES: bread wheat with rye translocation; Rye NO: bread wheat without rye translocation.

|  | **Bread wheat** | | | | **Durum wheat** | | **Tritordeum** | |
| --- | --- | --- | --- | --- | --- | --- | --- | --- |
|  | **Rye YES** | | **Rye NO** | |  | |  | |
|  | **Mean** | **Range** | **Mean** | **Range** | **Mean** | **Range** | **Mean** | **Range** |
| **Alpha-gliadin amplicon** |  |  |  |  |  |  |  |  |
| Number of amplicons | 31.0 | (22-40) | 34.2 | (23-45) | 33.0 | (32-34) | 31.8 | (31-32) |
| Number putative genes | 24.5 | (21-30) | 26.4 | (21-32) | 21.0 | (20-22) | 19.8 | (19-20) |
| Number pseudogenes | 6.5 | (1-12) | 7.8 | (1-13) | 12.0 | (12-12) | 12.0 | (12-12) |
| Ratio pseudogenes/p. genes | 0.26 | (0.05-0.44) | 0.28 | (0.05-0.48) | 0.57 | (0.55-0.60) | 0.61 | (0.60-0.63) |
| Total number of epitopes | 49.9 | (35-77) | 57.5 | (36-80) | 46.5 | (46-47) | 42.6 | (42-43) |
| **Gamma-gliadin/secalin amplicon** |  |  |  |  |  |  |  |  |
| Number of amplicons | 15.0 | (12-20) | 17.3 | (13-22) | 8.0 | (8-8) | 8.4 | (8-10) |
| Number putative genes | 11.5 | (8-16) | 14.3 | (10-18) | 5.0 | (5-5) | 5.0 | (5-5) |
| Number pseudogenes | 3.5 | (3-4) | 3.0 | (1-5) | 3.0 | (3-3) | 3.4 | (3-5) |
| Ratio pseudogenes/p. genes | 0.32 | (0.21-0.50) | 0.21 | (0.06-0.38) | 0.60 | (0.60-0.60) | 0.68 | (0.60-1.00) |
| Total number of epitopes | 73.0 | (51-125) | 123.0 | (90-156) | 54.0 | (54-54) | 58.8 | (54-78) |

**Supplementary Table 6.** Abundance of CD epitopes in alpha-gliadins’ putative gene amplicons.

**Supplementary Table 7.** Abundance of CD epitopes in gamma-gliadins/secalins’ putative gene amplicons.

**Supplementary Table 8.** Abundance of alpha-gliadins’ amplicons ‘types’ for each genotype. Each ‘type’ was established by the number of epitopes matched in the putative gene amplicon peptide sequences.

**Supplementary Table 9.** Abundance of gamma-gliadins’ amplicons ‘types’ for each genotype. Each ‘type’ was established by the number of epitopes matched in the putative gene amplicon peptide sequences.

**Supplementary Table 10.** The log_2_(FC) of the abundance for all the CD epitopes and p31-41 peptide variants found in the alpha-gliadins amplicons for each genotype. The mean of the log_2_(FC) for all the genotypes was calculated for each variant. The variants with the mean higher than zero are indicated in bold. FC: fold-change.
