# Supplementary figures and images for: Unraveling the celiac disease-related immunogenic complexes in a set of wheat and tritordeum genotypes: implications for low-gluten precision breeding in cereal crops
Source: Front Plant Sci. 2023 May 11;14:1171882. doi: 10.3389/fpls.2023.1171882 (PMC10210591; doi:10.3389/fpls.2023.1171882)

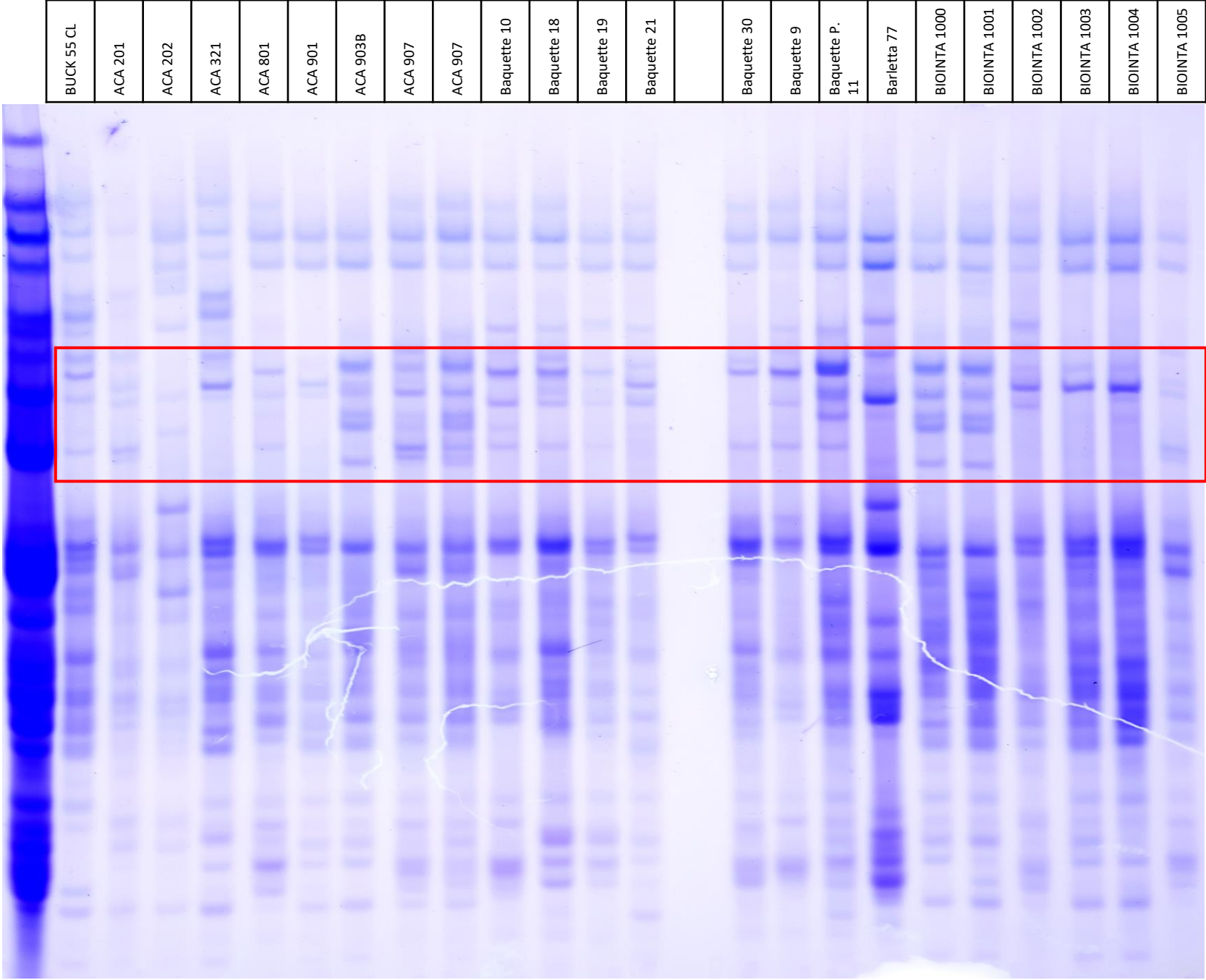

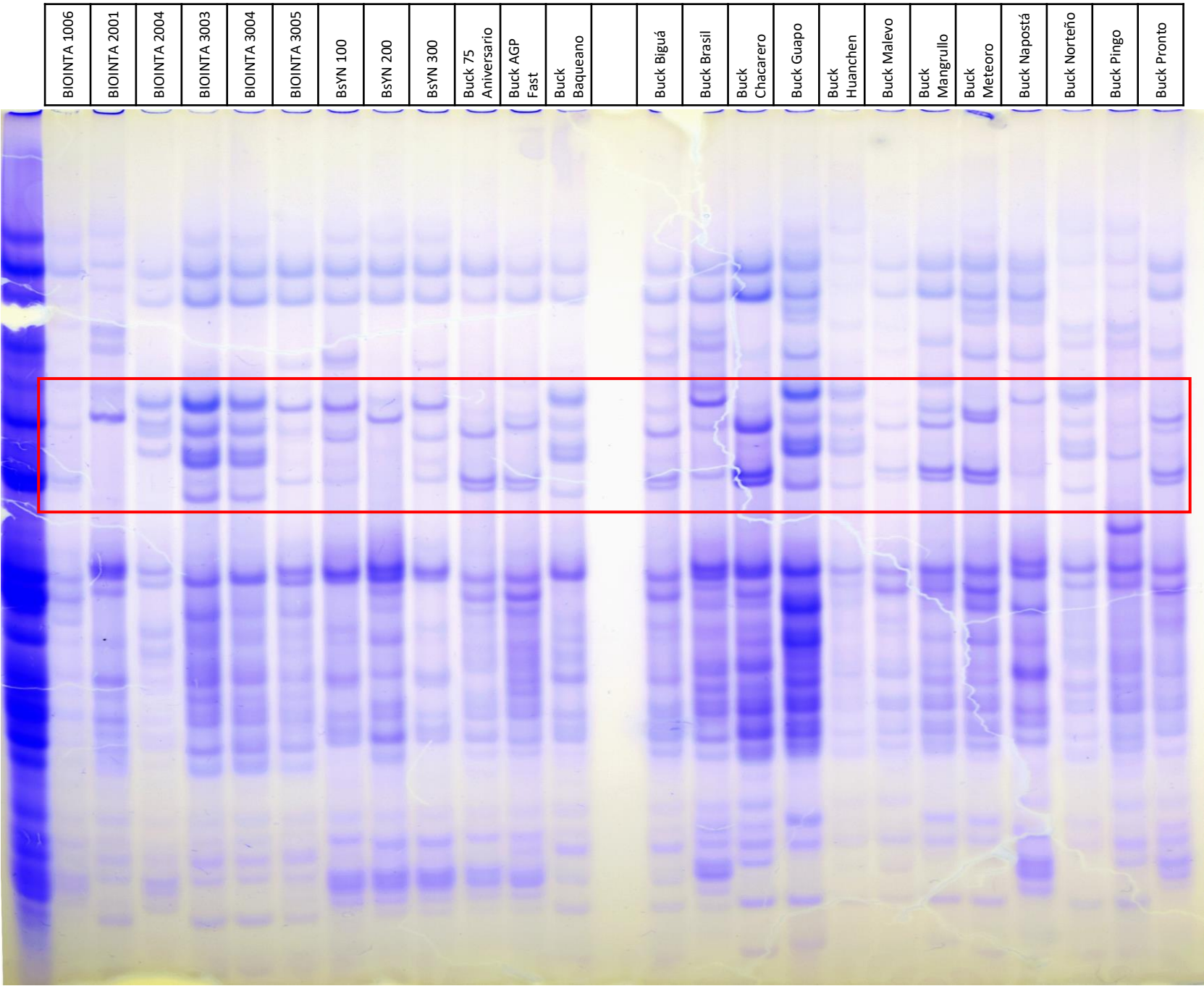

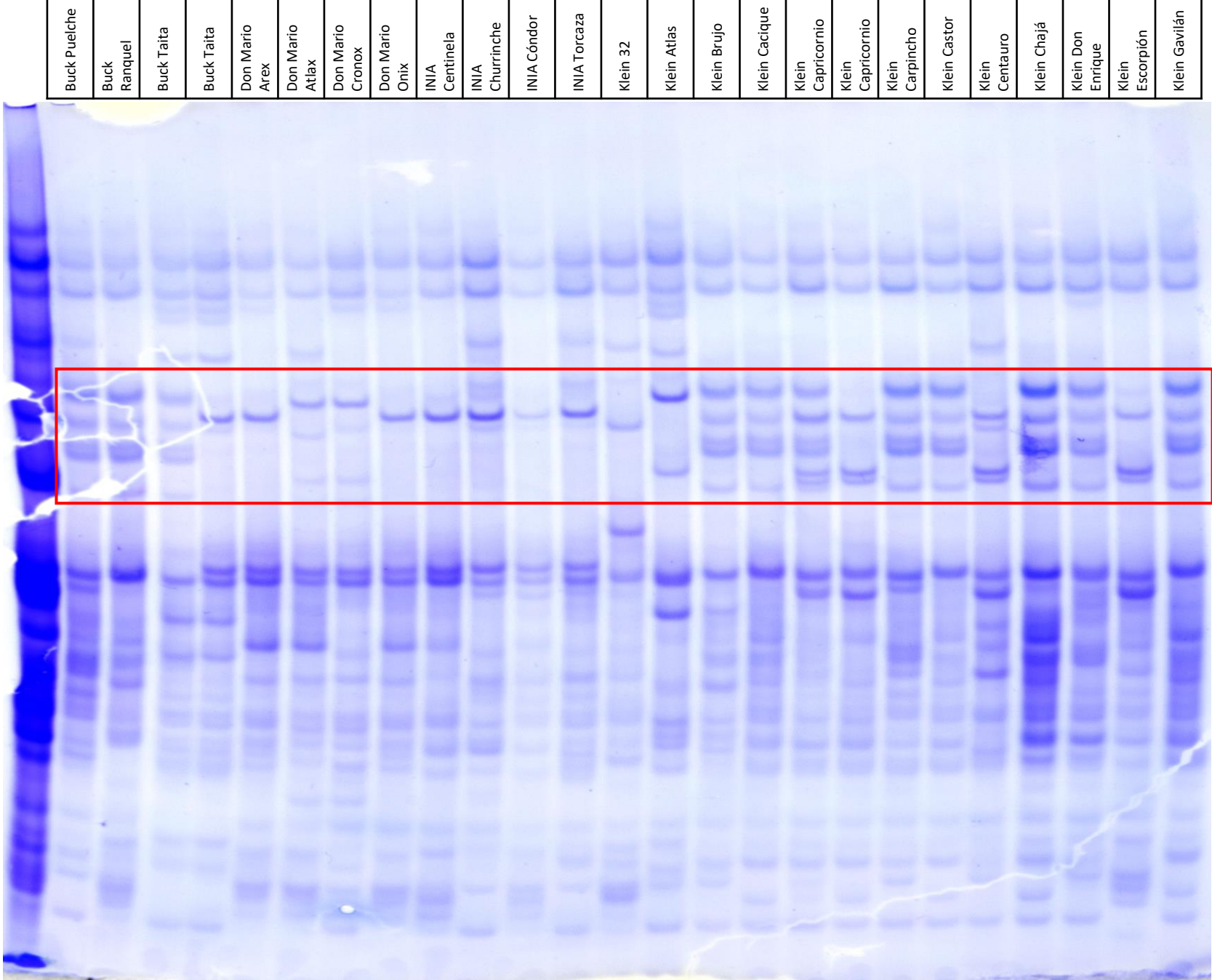

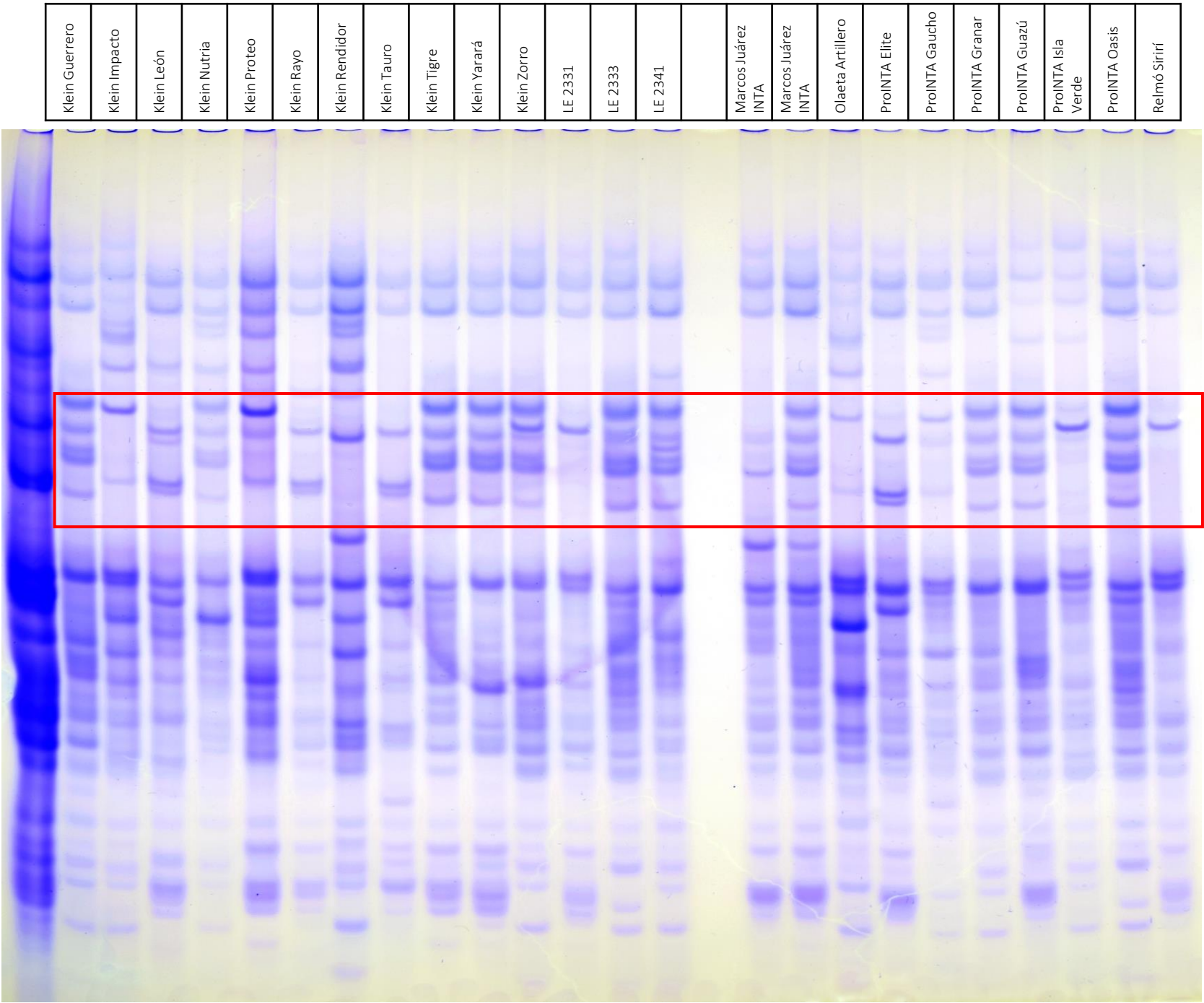

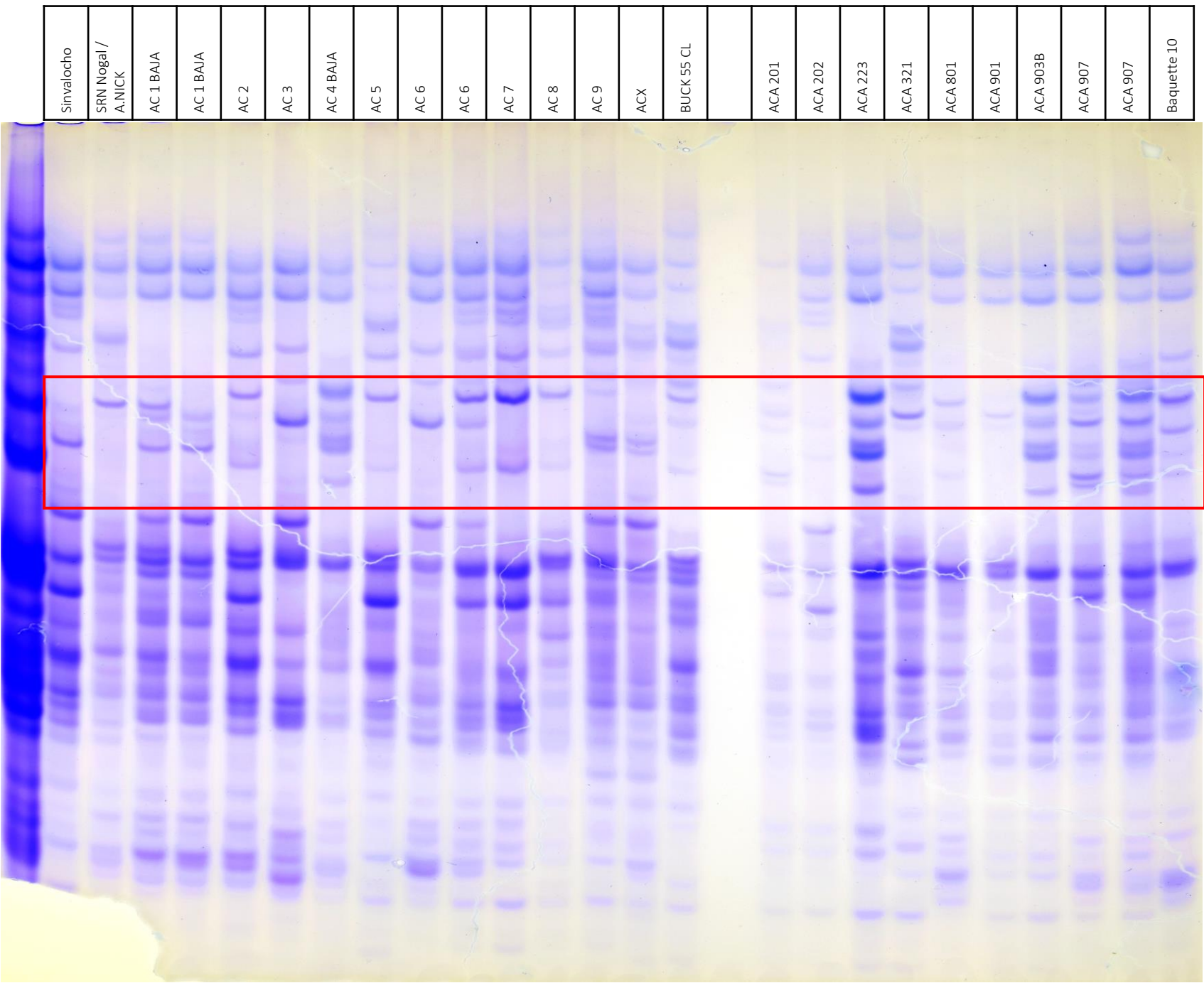

Supplement: Supplementary file 1 [file DataSheet_1.zip › Figure S2.pdf]
